# Restrictive Legislation

**Published:** 1914-10

**Authors:** 


### Restrictive Legislation

The time is ripe to demand a serious and systematic inquiry into legislation affecting the practice of medicine, and the enunciation of general principles and limitations. It is a serious embarassment of our government that an act may be perfectly legal in one state and not in another and that the enactment of interstate regulations by the general government may, instead of merely bridging over a gap between two authorities, render an act criminal because it involves a passage from one state to another while it would not be so if completely executed in either state. Without venturing to suggest means, we hold that something should be done to remedy these conditions. Unless there are peculiar local conditions which may render an act harmful in one community and not in another, there should be a common legal status of all acts involving principles of right and wrong throughout the whole country.

That certain legal restrictions should envelope the practice of medicine requires no argument. That these restrictions may become so numerous and so complicated that they impose serious obligations on any conscientious and intelligent man is, however, a present danger and a danger of this nature is not only an unwarranted affront to and imposition upon a well meaning profession but a serious handicap upon the power of the profession to serve the public.

We have reached a state of economic development in which too many persons have too much leisure, and a state of ethical development in which this leisure is spent by many, not in self indulgence but in endeavoring to reform and improve the condition of their neighbors. This tendency is laudable but somewhat dangerous, as altruism cannot appreciate the conditions and desires of one's neighbor quite as well as egoism can for itself. When altruism reaches the point of legislating its kind thoughts upon the actions of its neighbor and finds other expressions of altruism ready to combine with it to carry such legislation by a majority of legislators, the danger becomes increased.

It is a time-honored legal principle that ignorance of the law is no excuse. But it should be remembered that this principle implies that the law is in accordance with common sense, that it represents a general principle of right or wrong of a commonly accepted way of obviating a difficulty or that, if arbitrary and local, it is well understood by a permanent local population. When in passing from one city to another of the same state, without radical differences in population or local conditions, the stranger finds that differences of law exist; when in the same city, he is held up by a policeman and threatened with arrest for doing what he was told to do by another policeman a few days previously; when what amounts practically to legislation can be formulated overnight by the police authorities, knowledge of the law becomes an impossibility.

Let us consider some special points of medical legislation. It must be admitted that the use of narcotics is objectionable but their occasional administration by a physician is necessary. If we regard the matter broadly, there are very few drugs and very few imponderable means of therapy which are not more or less objectionable and which may not even be dangerous under certain conditions. Why specify certain drugs for which a special system of bookkeeping is required, which may not be sent by messenger or administered by the physician's assistant? Such a course hampers the physician's efficiency, establishes the precedent that he is to be supervised by some one else, perhaps not at all competent, at any rate not possessed of definite information as to the particular circumstances, diverts his attention from the main issue of using his best judgment in the interests of his patient. If any one could give an accurate definition of a narcotic and absolutely distinguish between narcotic and non-narcotic drugs, the case might be different.

Many narcotic drugs are habit forming and vice versa, but no definite classification can be made of habit forming drugs. When cocaine first came to the notice of the profession, one of the strongest claims made for it was that there was no danger of establishing the habit. There are doubtless many other drugs not as yet familiar to the laity which may subsequently prove to be habit-forming. Strychnine, nitroglycerin, chocolate, sodium bicarbonate, magnesium hydroxid, magnesium oxid, sodium phosphate, phenolphthalein, cod liver oil, pepsin, charcoal, and many other substances, more or less active and, therefore more or less harmful, may be used habitually and may, directly or indirectly, positively or negatively, do considerable harm.

Just at present, there is a wave of hysteria regarding mercuric chloride and resulting from the hysteria, so much publicity that there is an excess of use of this substance for suicide. It does not seem to have occurred to our legislators that practically any other soluble mercuric salt is as dangerous or that, if they prescribe restrictions to its use irrespective of the convenience of the physician, there are many other poisons that should be placed under the same restrictions. We have seen serious toxic symptoms from peppermint and there are dozens of common plants, growing in accessible places or to be found on the shelves of drug stores which can be used for suicidal purposes just as well. In fact, practically every substance that has therapeutic power possesses danger, even to life, if taken in sufficient amount, and for a sufficient time.

We believe that there are on the statute books, though not particularly in evidence at present, special laws regarding abortifacient drugs. Yet the medical literature abounds with articles claiming that these drugs, seriatim, are not specifically abortifacient, even that they may cause death without producing abortion.

It seems to us that some general method can be evolved to prevent the careless or suicidal or homicidal use of drugs by ignorant persons, without entangling the medical profession in a snarl of red tape, and without arbitrarily establishing legal classifications and lists of drugs which are not supported by actual facts. We hold that, with due allowance for unfamiliarity with new drugs and new imponderables, there is no therapeutic agency which may not be used safely and to the advantage of the patient, by a properly trained physician, in proper dose and under proper conditions. We hold that there is no chemic substance and no force which, ignorantly, carelessly and injudiciously used, may not produce serious harm. Even water comes under this statement. A poultice may cause death. Practically every form of energy may be lethal. We feel that, after subjecting a candidate to a rigorous examination for license, he should be allowed to practice his profession without molestation or restriction until his license is removed for just cause. This is a strong statement but we consider that, in the long run, it is a safer policy for the public than to attempt to specify agencies which he may use under ordinary general laws and those which he may use only under special prohibitive regulations.

Similarly, we hold that equally general regulations should be made with regard to pharmacists, nurses and others who assist the medical profession in various ways and that the same broad policy should apply to the public, adopting the general principle in the case of this last class, that it is not competent to select its own remedial agents, pharmacologic or otherwise. It must be confessed that this last principle is not susceptible of perfect application. There is an abundance of chemicals and forces employed in the arts and industries, including the domestic ones, which may be put to dangerous uses. Dense ignorance and obstinate desire for suicide or homicide cannot well be thwarted. Poisoning can be accomplished by soda, baking powder, turpentine, gasoline, natural and artificial gas, by such common weeds as deadly nightshade and, even if the last were exterminated by potato skins which contain the same active principle. In fact, we may go still farther and claim that neither legislatures nor amateur societies for eliminating this and that evil and danger can supersede the necessity for an individual conscience and an individual common sense. And this is only another way of saying that we might just as well live up to the theory that this is a free country and stop the tendency to what is called reform before we reach a point that will make an absolute monarchy under military law look like a riot of license by contrast.

